# Complete Response to Low-Dose Fam-Trastuzumab Deruxtecan in Recurrent Endometrial Cancer with Liver and Peritoneal Metastases: A Case Report

**DOI:** 10.1159/cro/abzag048

**Published:** 2026-09-10

**Authors:** Fang-Yu Wang, Cheng-Han Wu, Sung-Chao Chu, Chiu-Hsuan Cheng, Yen-Chang Chen, Han-Kuang Hsieh, Tso-Fu Wang

**Affiliations:** a Division of Hematology and Oncology, Department of Internal Medicine, Hualien Tzu Chi Hospital, Buddhist Tzu Chi Medical Foundation, Hualien, Taiwan; b School of Medicine, Tzu Chi University, Hualien, Taiwan; c Department of Anatomical Pathology, Hualien Tzu Chi Hospital, Buddhist Tzu Chi Medical Foundation, Hualien, Taiwan

**Keywords:** Endometrial carcinoma, HER2 amplification, Trastuzumab deruxtecan, Antibody-drug conjugates, Dose optimization

## Abstract

**Introduction:**

While fam-trastuzumab deruxtecan (T-DXd) has demonstrated significant efficacy in HER2-expressing solid tumors, its optimal dosing and tolerability in elderly or frail patients remain poorly defined. Evidence supporting upfront dose de-escalation in gynecologic malignancies is limited.

**Case Presentation:**

We report a 73-year-old frail woman with recurrent, metastatic endometrial carcinoma featuring hepatic and peritoneal metastases, HER2 immunohistochemistry 3+, and high-level *ERBB2* amplification. Following progression on multiple platinum-taxane regimens, bevacizumab, surgery, hormonal therapy, and radiotherapy, the patient was treated with a fixed, reduced dose of T-DXd at 100 mg every 2 weeks (2.27 mg/kg per administration, approximately 63% of the standard-dose intensity). She achieved a complete radiological response by 6 months, with clinical improvement and no remarkable adverse events, sustaining a progression-free survival exceeding 16 months at last follow-up.

**Conclusion:**

This case illustrates a favorable response to dose-reduced T-DXd in a frail patient with strongly HER2-driven endometrial cancer, raising the possibility of individualized dosing strategies that warrant evaluation in larger prospective or retrospective cohorts.

## Introduction

Recurrent and metastatic endometrial cancer remains a therapeutic challenge, particularly in patients who progress after platinum-based chemotherapy and anti-angiogenic therapy. A contemporary real-world cohort indicates a median progression-free survival (PFS) of only 7.4 months (95% CI: 6.2–8.0) and a median overall survival of 11.0 months (95% CI: 9.9–12.3) following first-line chemotherapy failure [1]. Among these tumors, a distinct subset – particularly high-grade or recurrent disease – harbors HER2 overexpression or *ERBB2* amplification, representing a promising target for antibody-drug conjugates. Although fam-trastuzumab deruxtecan (T-DXd) has demonstrated robust efficacy across HER2-expressing solid tumors, data regarding its efficacy, optimal dosing, and tolerability in endometrial cancer remain limited, particularly among elderly and frail patients with extensive prior treatment exposure.

Here, we present the case of a 73-year-old woman with heavily pretreated, recurrent HER2-amplified endometrial adenocarcinoma with hepatic and peritoneal metastases who achieved a complete radiologic response with a fixed, reduced dose of T-DXd (100 mg [2.27 mg/kg] every 2 weeks, representing approximately 63% of the standard-dose intensity), sustaining a PFS exceeding 16 months at the latest follow-up. This case illustrates a favorable response to dose-reduced T-DXd in frail patients, raising the possibility of individualized dosing strategies in advanced endometrial cancer that warrant further evaluation. This case report was prepared in accordance with the CARE Guidelines, and the completed CARE Checklist is provided online as online supplementary material (for all online suppl. material, see https://doi.org/10.1159/cro/abzag048).

## Case Presentation

A 73-year-old woman with chronic hepatitis B had a remote history of rectal adenocarcinoma (pT3N2M1, with liver metastases) diagnosed in 2008 at the age of 56, treated with low anterior resection and partial hepatectomy, followed by postoperative chemoradiotherapy and systemic chemotherapy, achieving a 10-year disease-free interval. In June 2019, she developed a second primary malignancy in the uterus, classified as FIGO stage IB (pT1b), grade 2 endometrioid adenocarcinoma. She was treated with hysterectomy and bilateral salpingo-oophorectomy without adjuvant therapy, remaining disease-free for 2.5 years.

In late December 2021, imaging revealed new hepatic, presacral, and peritoneal lesions. Notably, all these and subsequent metastatic recurrences were pathologically and immunohistochemically (CK7+/PAX8+; CK20−/CDX2−) confirmed to be of primary endometrial origin, with no evidence of rectal cancer relapse. Systemic anticancer treatment commenced in March 2022, with all details summarized in Table 1. First-line carboplatin/paclitaxel (March–October 2022) achieved complete radiologic response, followed by UFUR (tegafur-uracil) maintenance.

**Table 1.** Treatment timeline

Date	Disease status	Treatment	DFS/PFS
June 2019	Diagnosis of endometrial carcinoma	Hysterectomy and bilateral salpingo-oophorectomy	DFS: 2.5 years
March 2022	Hepatic and peritoneal recurrence	8 cycles of carboplatin/paclitaxel	PFS: 13 months
October 2022	Radiologic CR	UFUR (tegafur-uracil) maintenance	
April 2023	Hepatic and peritoneal progression	12 cycles of platinum/paclitaxel + bevacizumab	PFS: 19 months
April 2024	Good PR with residual liver tumors	Partial hepatectomy	
		Megestrol acetate and tamoxifen maintenance	
November 2024	Hepatic and presacral recurrence	Radiotherapy (50 Gy in 25 fractions)	​
March 2025	Hepatic progression	T-DXd 100 mg every 2 weeks	PFS: 16 months, ongoing

CR, complete response; DFS, disease-free survival; PFS, progression-free survival; PR, partial response; T-DXd, fam-trastuzumab deruxtecan.

After a 13-month progression-free period, new hepatic and peritoneal lesions appeared in April 2023, prompting re-treatment with carboplatin/paclitaxel combined with bevacizumab. It yielded a partial response after 6 cycles, but was complicated by grade 3 myelosuppression and recurrent urinary tract infections. Small residual liver tumors were resected in April 2024 (segment 2/3 hepatectomy), followed by hormonal maintenance with megestrol acetate and tamoxifen. However, hepatic and presacral recurrence developed 7 months later in October 2024, and disease continued to progress despite local radiotherapy.

By February 2025, computed tomography (CT) demonstrated at least four enlarging right hepatic metastases (Fig. 1). Biopsy showed HER2 IHC 3+ expression (Fig. 2), and NGS revealed high-level *ERBB2* amplification (estimated copy number, 85), along with a *TP53* mutation, microsatellite-stable status, and no pathogenic *BRCA* variant. Given her advanced age, frailty, extensive prior treatment, and financial constraints, a reduced fixed dose of fam-T-DXd, 100 mg in a 44-kg patient (2.27 mg/kg) every 2 weeks, was selected through shared decision-making. This initial dose corresponds to 63% of the standard-dose intensity of 5.4 mg/kg every 3 weeks.

**Fig. 1.**
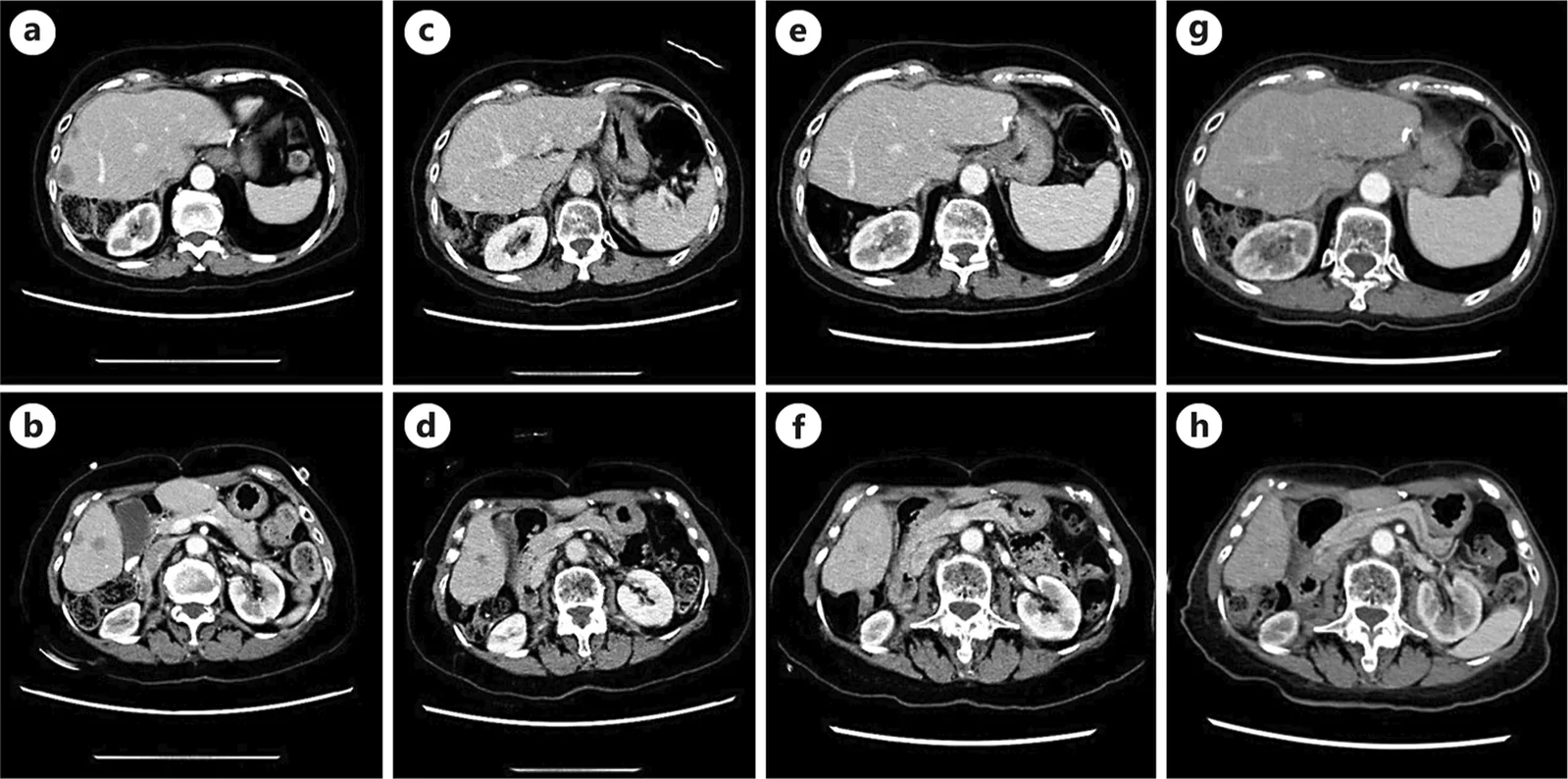
Serial CT images during treatment with fam-T-DXd. **a**, **b** February 2025: baseline imaging showed multiple measurable hepatic metastases before T-DXd. **c**, **d** May 2025: marked reduction in the size of hepatic metastases after approximately 2 months of T-DXd. **e**, **f** September 2025: disappearance of all hepatic target and nontarget lesions, meeting RECIST version 1.1 criteria for CR. **g**, **h** July 2026: continued absence of all hepatic lesions.

**Fig. 2.**
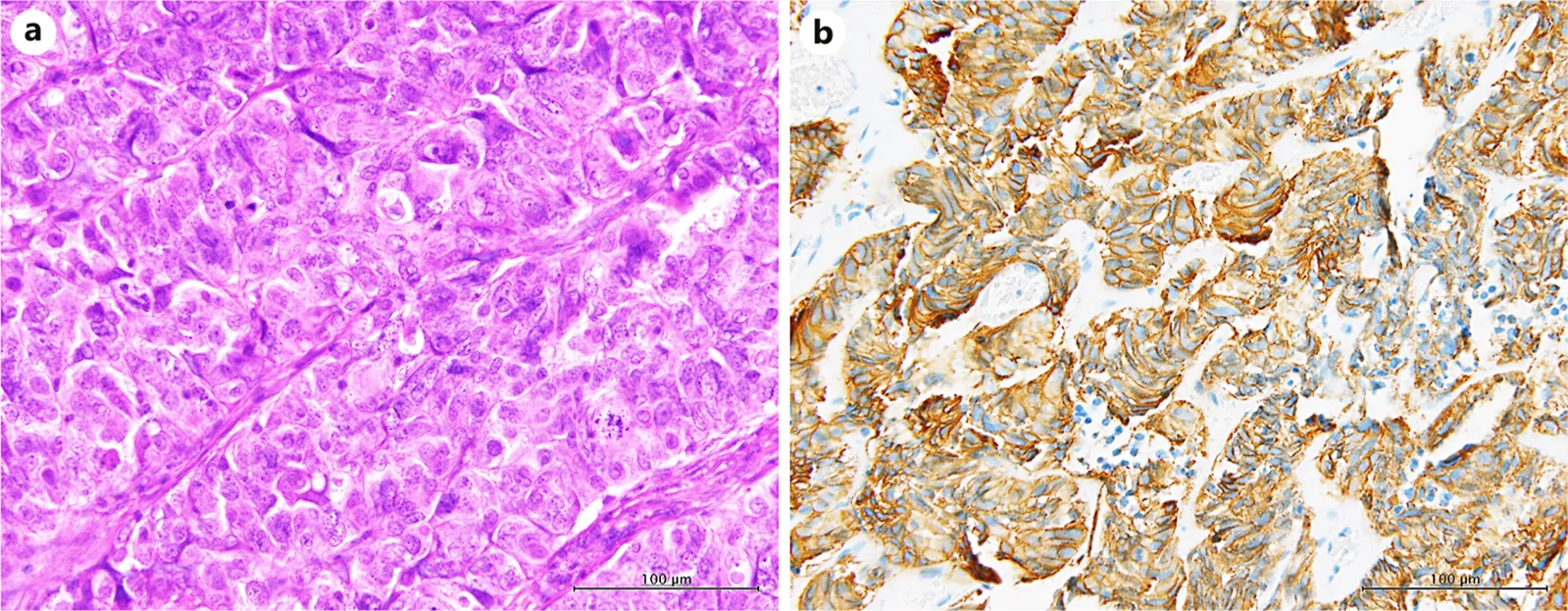
Histological and HER2 immunohistochemical findings in metastatic endometrioid carcinoma. **a** Hematoxylin and eosin stain showed high-grade adenocarcinoma with marked nuclear atypia and brisk mitotic activity. **b** HER2 immunohistochemistry was scored as 3+ because of intense complete or basolateral/lateral membranous staining in more than 10% of viable tumor cells.

T-DXd treatment commenced on 6 March 2025. Follow-up CT showed marked hepatic tumor regression by May 2025 and complete resolution of all target and nontarget lesions by September 2025, meeting RECIST v1.1 criteria for complete response (CR). The dosing interval was subsequently extended to 100 mg every 3 weeks in December 2025. As of her most recent CT scan in July 2026 (Fig. 1), she remains in complete radiologic remission, with a PFS exceeding 16 months. Throughout treatment, the patient underwent regular safety monitoring, including complete blood counts and biochemistry panels before each cycle, LVEF assessment by echocardiogram every 6 months, and CT scans performed concurrently with tumor response assessment every 3 months, together with pulse oximetry and symptom screening for pulmonary toxicity at each visit. She remains clinically well and asymptomatic, with preserved performance status and organ function. Laboratory results remained stable throughout treatment, with only mild anemia (hemoglobin ∼11 g/dL; CTCAE grade 1) observed; no neutropenia, thrombocytopenia, or hepatic/renal dysfunction was documented. LVEF remained within normal limits, and no clinically significant interstitial lung disease or cardiac dysfunction was observed.

## Discussion

This case documents a complete and durable response to T-DXd in recurrent *ERBB2*-amplified metastatic endometrial carcinoma after multiple lines of systemic and local therapy. This favorable response is consistent with emerging evidence that HER2-expressing gynecologic tumors – particularly endometrial cancer – can be highly sensitive to T-DXd. In DESTINY-PanTumor02, among 40 enrolled patients with endometrial cancer, the objective response rate (ORR) was 57.5%, with a median PFS of 11.1 months and overall survival of 26.0 months [2]. However, the principal novelty of our case lies not merely in the response to T-DXd itself, but in the maintenance of antitumor activity with an upfront fixed-dose schedule delivering only approximately 63% of the standard-dose intensity. Clinical trials have established the efficacy of T-DXd at a standard dose of 5.4 mg/kg every 3 weeks in breast cancer and 6.4 mg/kg every 3 weeks in gastric cancer [3, 4]. Similarly, currently available gynecologic case reports and small real-world series have largely described outcomes using standard starting doses. Approved dose modification schemes for T-DXd generally involve stepwise reductions from 5.4 mg/kg to 4.4 mg/kg, and subsequently to 3.2 mg/kg every 3 weeks, following toxicity [3]. In the present case, the upfront dose applied was 2.27 mg/kg every 2 weeks – representing only 63% of the standard-dose intensity – yet sustained a durable response.

Notably, the tumor in our patient harbored a markedly high estimated *ERBB2* copy number of 85, which may account for the exceptional response observed despite the use of a reduced T-DXd dose intensity. High-level *ERBB2* amplification likely translates into a high density of cell-surface HER2 receptors and a stronger tumor dependence on HER2-driven signaling, potentially permitting substantial antibody binding, internalization, and payload delivery even when circulating drug exposure is reduced. This is consistent with emerging data across HER2-targeted antibody-drug conjugate trials suggesting that the magnitude of HER2 expression and amplification correlates with treatment response. In the exploratory biomarker analysis of DESTINY-Gastric01, patients with high adjusted plasma *ERBB2* copy number achieved a higher ORR than those with lower plasma copy number (78.6% vs. 34.3%). Although this plasma-based analysis cannot be directly compared numerically with our patient’s tissue-based *ERBB2* copy number of 85, the same study reported that plasma *ERBB2* copy number correlated with tissue HER2 status, supporting the broader principle that greater HER2 amplification is associated with greater T-DXd sensitivity [5]. Similarly, in the exploratory biomarker analysis of the endometrial cancer cohort from DESTINY-PanTumor02, ORR was higher among patients with detectable tissue or plasma HER2 amplification (84.6% and 90.9%, respectively) compared with those without detectable amplification (45.0%) [6]. However, this dose-response relationship has been characterized under standard-dose T-DXd regimens, and its applicability to reduced-dose strategies in endometrial cancer remains unestablished. This biomarker-driven hypothesis therefore remains speculative in our setting and suggests that our findings may not be generalizable to all HER2-positive endometrial cancers, particularly those with lower level amplification, while also underscoring the potential rationale for biomarker-guided, individualized dosing strategies warranting further validation.

Beyond high-level target amplification at the biopsied site, the pharmacologic properties of T-DXd itself may further explain the extensive and durable response observed across multiple metastatic compartments in this patient. T-DXd has a drug-to-antibody ratio of approximately 8 and a cleavable linker that releases a membrane-permeable topoisomerase I inhibitor payload. The resulting bystander effect can kill neighboring tumor cells with lower or heterogeneous HER2 expression [4]. This property may be particularly relevant in our patient, who harbored spatially separated hepatic and peritoneal metastases, sites where HER2 expression can vary considerably in endometrial cancer. Nevertheless, HER2 heterogeneity was not directly assessed across all metastatic sites in this case, and the CR cannot be attributed to the bystander effect alone.

Treatment-related toxicity is a significant concern in T-DXd therapy, particularly in elderly or frail patients, and is generally dose-dependent. Key toxicities include interstitial lung disease, occurring in 10–15% of patients, along with myelosuppression and gastrointestinal adverse effects [3, 7, 8]. In DESTINY-PanTumor02, 35% of patients with endometrial cancer experienced grade ≥3 adverse events, and 32.5% required dose modifications; no endometrial-specific efficacy or toxicity analyses were provided for older or frail subgroups. Emerging real-world data in HER2-positive breast cancer suggest that dose de-escalation may be a pragmatic strategy in this population. In the TREX-Old registry of elderly patients with breast cancer [9], 30% of patients started T-DXd at a reduced dose, and 30% required a subsequent dose adjustment during treatment. In a broader age-specific pooled analysis of the DESTINY-Breast trials, patients older than 65 years showed a safety profile similar to that of younger patients, albeit with a trend toward greater severity of adverse events [10]. To our knowledge, no comparable cohort has specifically evaluated dose de-escalation strategies for T-DXd in frail patients with gynecologic malignancies. The favorable toxicity profile observed with low-dose T-DXd in this patient is therefore noteworthy, allowing meaningful clinical improvement without treatment interruption – in marked contrast to her prior therapies, which were complicated by grade 3 myelosuppression, infections, and diarrhea.

Conventional second-line chemotherapy for recurrent endometrial cancer generally produces low response rates, typically ranging from 5 to 15%, with short disease control [1, 11]. The CR and favorable tolerability observed in this heavily pretreated, frail patient are therefore clinically notable and support further investigation of biomarker-guided, vulnerability-adapted dosing strategies for T-DXd in advanced endometrial cancer.

## Study Limitations

This report has several limitations. First, it describes a single patient, and the findings are hypothesis-generating rather than confirmatory of the efficacy or safety of reduced-dose T-DXd. Second, the absence of pharmacokinetic sampling precluded confirmation of actual drug exposure at the reduced dose. Third, although PFS currently exceeds 16 months, longer follow-up is needed to confirm durability of response. Finally, given the patient’s markedly high-level *ERBB2* amplification, these findings may not be generalizable to HER2-low or *ERBB2*-nonamplified endometrial cancers. Prospective studies incorporating pharmacokinetic sampling, geriatric assessment, tumor HER2 quantification, and systematic toxicity surveillance are warranted.

## Conclusion

We present a heavily pretreated older woman with HER2 IHC 3+, high-level *ERBB2*-amplified recurrent endometrial cancer who achieved a durable complete radiographic response with fixed-dose T-DXd 100 mg every 2 weeks, corresponding to approximately 63% of the standard-dose intensity. This case offers preliminary real-world insight into the clinical efficacy, tolerability, and potential for individualized dosing of T-DXd in vulnerable populations with advanced endometrial cancer.

## Statement of Ethics

Written informed consent for publication of the clinical information and accompanying radiologic and histopathologic images was obtained from the patient on February 24, 2026. The study report was reviewed and approved by Research Ethics Committee of Tzu Chi General Hospital, Buddhist Tzu Chi Medical Foundation (Approval No. CR115-007). The CARE Checklist was completed and is provided as online supplementary material.

## Conflict of Interest Statement

The authors declare that they have no known competing financial interests or personal relationships that could have appeared to influence the work reported in this paper.

## Funding Sources

The authors received no funding for the research, authorship, and preparation of this article.

## Author Contributions

Fang-Yu Wang: conceptualization, data curation, investigation, and writing – original draft. Cheng-Han Wu, Sung-Chao Chu, and Chiu-Hsuan Cheng: writing – review and editing. Yen-Chang Chen: pathology review and writing – review and editing. Han-Kuang Hsieh: clinical data review and writing – review and editing. Tso-Fu Wang: conceptualization, investigation, supervision, and writing – original draft and review.

## Data Availability

All data relevant to this case are included in this article and its online supplementary material. Additional de-identified information may be available from the corresponding authors subject to institutional and ethical restrictions.
